# Effects of the COVID-19 pandemic on perinatal outcomes: a retrospective cohort study from Turkey

**DOI:** 10.1186/s12884-021-04349-5

**Published:** 2022-01-20

**Authors:** Siddika Songül Yalçin, Perran Boran, Başak Tezel, Tuba Esra Şahlar, Pınar Özdemir, Bekir Keskinkiliç, Fatih Kara

**Affiliations:** 1 Unit of Social Pediatrics, Department of Pediatrics, Faculty of Medicine, Hacettepe University, Ankara, Turkey; 2 Division of Social Pediatrics, Department of Pediatrics, School of Medicine, Marmara University, İstanbul, Turkey; 3 Department of Child and Adolescence Health, General Directorate of Public Health, Ministry of Health, Ankara, Turkey; 4 Department of Women’s and Reproductive Health, General Directorate of Public Health, Ministry of Health, Ankara, Turkey; 5 Department of Biostatistics, Faculty of Medicine, Hacettepe University, Ankara, Turkey; 6 General Directorate of Public Health, Ministry of Health, Ankara, Turkey

**Keywords:** COVID-19, Preterm birth, Mode of delivery, Birth weight, Pregnancy, Region

## Abstract

**Background:**

Lockdowns, pregnant women’s fear from hospitalization in addition to uncertainties about appropriate birthing practices at the beginning of the pandemic may have affected the health outcomes of mother-infant couples. We aimed to explore whether pregnancy outcomes including the rates of cesarean delivery (CS), preterm, and low birth weight (LBW) births have changed during the pandemic period compared with the pre-pandemic period.

**Methods:**

We applied a population-based retrospective cohort, before-after approach in 2020 vs. similar calendar months in 2019 for five periods [Jan-Feb (pre-pandemic); March–May (1st wave and lockdown); June–August; September–October; November–December (2nd wave and lockdown)]. The data was modelled through multiple logistic regressions using key outcomes; CS, preterm, and LBW births as the dependent variables, and adjustments were made for independent variables in SPSS software. We evaluated the modification of years by periods by adding interaction term (yearXperiod) to the model.

**Results:**

The rate of CS in hospital births increased from 57.7% in 2019 to 60.2% in 2020. CS rates were significantly increased during the 3rd and 4th periods. The overall preterm rate was 11%. When singleton pregnancies were considered, adjusted multivariable analyses showed a decrease in preterm proportions during all time periods with respect to the pre-pandemic period. The percentage of LBW was 7.7% during the pandemic period and was found to be significantly reduced compared to the pre-pandemic period. There was a significant reduction in LBW rates in all periods except the second lockdown period.

**Conclusions:**

Our findings suggested significant reductions in preterm and LBW births possibly due to the indirect effects of the pandemic. Moreover, strategies need to be considered to address the increased CS rates and shifting of maternity service utilization to private facilities.

**Supplementary Information:**

The online version contains supplementary material available at 10.1186/s12884-021-04349-5.

## Background

Turkey reported its first COVID-19 case on March 10, 2020. Flexible working hours and working home-office were applied as of March 16, and pregnancy women over 24 weeks of gestational age have been granted on administrative leave in Turkey [1, 2]. A curfew has been implemented for those over the age of 65 on March 21st, and those under the age of 20 on April 3rd. As of April 11, curfews and quarantine measures were implemented on weekends for 30 metropolitan cities except for certain sectors. A nationwide lockdown was implemented on 16th May 2020 for 4 days. As of June 1st, a stepwise de-escalation period was implemented, and public places reopened as part of the normalization process. As of mid-November, curfews were re-implemented [2]. The two-wave pattern of reported cases was seen, the first wave in spring and the second wave in autumn.

The COVID-19 pandemic can impact reproductive and perinatal health both directly through infection itself but also indirectly as a consequence of changes in health care, social policy, or social and economic circumstances, nationwide lockdowns [3, 4]. It has been reported that pregnant women were afraid to visit hospitals for prenatal care [5]. Furthermore, lockdown measures might have influenced the prenatal visits. At the beginning of the COVID pandemic, there were no consensus and guidelines about proper management for birthing practices that reduce the spread of infection and improve the prognosis of health outcomes of mother-infant couples. It was reported that 13.7% of asymptomatic pregnant women admitted for delivery tested positive for COVID-19 [6] and women with COVID-19 were less likely to have a vaginal delivery [7, 8]. It was suggested that in the presence of COVID-19, the threshold for cesarean (CS) delivery should be lower than usual so that infection control procedures to minimize disease transmission can be more readily adhered [9]. Women exert extreme effort and frequently blow out their breath, cough, and shout which put the health care workers at risk. Based on the available evidence, the risk of neonatal COVID-19 infection, and maternal deaths are not increased when the mother gave birth through vaginal delivery. CS delivery should therefore be based on obstetric (fetal or maternal) indications and not COVID-19 status alone [8, 10, 11].

Pregnant women were exposed to stress during the pandemic, creating an adverse intrauterine environment which could result in shorter gestation and low birth weight (LBW) rates [12]. On the other hand, preliminary evidence has suggested a reduction, particularly in preterm and LBW births following COVID-19 prevention measures [13–15]. Yet others [16–19] demonstrated no changes in the incidence of preterm births. Findings remain unclear, and there is still a need for additional studies in different countries, across diverse demographic strata. Understanding of perinatal outcomes during the pandemic could guide policymakers in decision-making.

Increased pregnancy complications (preterm delivery 4.1%, miscarriage 2.2%) and CS (66.4%) were reported on pregnant women confirmed COVID-19 infection in a hospital based study from Ankara, Turkey [20]. A multicenter study in Turkey (*n* = 125) detected high frequencies of CS (71.2%), prematurity (26.4%), and low-birthweight infant rates (12.8%) in 125 newborns born to women infected with COVID-19 [21]. To the best of our knowledge, no national study covering all births has yet assessed pregnancy outcomes during the pandemic in Turkey. We aimed to explore whether pregnancy outcomes particularly CS, preterm, and LBW births have changed during the pandemic compared with pre-pandemic rates in Turkey.

## Methods

In this retrospective cohort study conducted in Turkey, before-after approach (in 2020 vs. similar calendar months in 2019) was applied to key pregnancy outcomes (CS delivery, preterm, and LBW rates) among births to estimate the effects of the COVID-19 pandemic.

The study was approved by Marmara University School of Medicine Ethical Committee (protocol no: 09.2020.699), the Turkish Ministry of Health, General Directorate of Public Health (protocol no: 67414668–604.02.99) and the Turkish Ministry of Health Services General Directorate Scientific Research Platform (18-June-2020).

The anonymized data including mothers’ information such as calendar month of birth, maternal age, parity, mode of delivery, prior CS, the onset of labor (spontaneous or induced), fetal presentation, and infant characteristics such as gestational age (in weeks), birth weight (in grams), sex, and health care service category of the delivery place was extracted from the electronic National “Birth Notification Registry system”. Birth Notification Registry system based on “erapor.saglik.gov.tr/portal” and had all hospital deliveries starting from 2018 [22]. The data in 2019 and 2020 included births that occurred in a health facility. However, births outside the health facility with the assistance of health personnel, and verbal declaration which included births that did not occur in a health facility or without the assistance of any health personnel were present in 2021. We excluded women with pregnancies where birth weight was < 500 g or women who delivered < 22 weeks of gestation. The data did not include information about the CS indications.

The level of 12 geographical regions was taken according to Nomenclature of Territorial Units for Statistics (NUTS)-1; Istanbul Region, West Marmara Region, Aegean Region, East Marmara Region, West Anatolia Region, Mediterranean Region, Central Anatolia Region, West Black Sea Region, East Black Sea Region, Northeast Anatolia Region, Central East Anatolia Region, Southeast Anatolia Region [23].

The study periods were categorized according to “five-time” frames. The first period included January, and February, 2 months before the first reported case (pre-lockdown months), the second period between March and May, corresponding to the first wave and lockdown period, the third period between June and August, the fourth period between September and October, the fifth period between November and December corresponding to the second wave and lockdown period.

Maternal age was categorized into 3 groups in completed years. Parity was defined as the number of previous pregnancies that crossed the age of viability. The last menstrual period was used to determine gestational age. Births were categorized according to the gestational age (weeks+days): extremely preterm (22 + 0–27 + 6), very preterm (28 + 0–31 + 6), moderate-to-late preterm (32 + 0–36 + 6), term (37 + 0–41 + 6) [17, 24]. Birth weight of infants was categorized as very low (VLBW, < 1500 g) and extremely low (ELBW, < 1000 g) regardless of gestational age [17]. Categories of health care services were divided into 3 groups; private, public, and university.

The 10-group classification system (TGCS) was applied to assess CS rates as recommended by World Health Organisation [25, 26]. TCGS is based on simple obstetrical parameters (parity, previous CS, gestational age, the onset of labour, fetal presentation, and the number of fetuses) and does not involve the indication for CS.

### Statistical analysis

The data were analyzed using IBM SPSS Statistics for Windows, Version 22.0. Armonk, NY: IBM Corp. Categorical data were expressed as numbers and percentages and were compared using the chi-square test and subgroup analysis were made with column proportions and Bonferroni method. Continuous variables were expressed as mean ± standard deviation. The data was modeled through logistic regressions using CS, preterm, and LBW births as the dependent variables, and adjustments were made for independent variables. Crude and adjusted Odds ratios (ORs) with 95% confidence interval (95%CI) were estimated using logistic regression methods. Two models were constructed for each different dependent variable [27]. Since we wanted to see the difference between the log-odds ratio comparing each period vs. the first period in 2020 and the log-odds ratio comparing each period vs. the first period in 2019, we evaluated the modification of years by periods by adding interaction term (yearXperiod) to the model. Independent variables, year, period, and their interaction were entered into the first model (Model 1).

To limit the influence of other determinants, we considered singletons in the cephalic presentation and primary CS cases for further analysis. Multiple logistic regression was used to analyze the association between CS rates and time periods with effect modification after adjustment for the year (2020 vs 2019), using time periods (month 3–5 vs 1–2; month 6–8 vs 1–2; month 9–10 vs 1–2; month 11–12 vs 1–2) as covariates (Model 1). Model 2 involved NUTS regions, maternal age, parity, gestational age, birth weight, the sex, and the onset of labor in addition to model 1.

Multiple logistic regression analysis for the determinants of preterm birth included cases with singleton deliveries. Model 1 was similar to CS analysis. Confounding factors in Model 2 were NUTS region, maternal age, parity, delivery type, the onset of labor, presentation, birth weight, sex, and Model 2 were also used to detect the association between time periods and preterm birth in spontaneous and medically induced deliveries (CS and induced).

Multiple logistic regression analysis for the determinants of LBW included cases with singleton births. Model 1 was similar to CS analysis and for Model 2 NUTS region, maternal age, parity, delivery type, the onset of labor, presentation, gestational age, sex were confounding factors.

All statistical tests were evaluated at an alpha level of 0.05.

## Results

A total of 2,219,914 hospital deliveries’ records were available in the database for the study period (1,141,385 in 2019 and 1,078,529 in 2020). The comparison between 2019 and 2020 was summarized in Table 1. Total deliveries were reduced during the first and second waves of the pandemic but were higher during the other periods.

**Table 1:** Comparison of maternal and pregnancy related characteristics between the pandemic year (January–December 2020) and the equivalent period preceding the pandemic in 2019

	Total births, 2019 *n* = 1,141,385 n (%)	Total births, 2020 *n* = 1,078,529 n (%)	*P*
**Periods**			< 0.001
Period 1 Jan-Feb	179,743 (15.7)^a^	172,071 (16.0)^b^	
Period 2 March–May	282,405 (24.7)^a^	261,500 (24.2)^b^	
Period 3 June–August	307,819 (27.0)^a^	295,911 (27.4)^b^	
Period 4 September–October	191,347 (16.8)^a^	183,478 (17.0)^b^	
Period 5 November–December	180,071 (15.8)^a^	165,569 (15.4)^b^	
Maternal age (years)*			< 0.001
< 20	39,994 (3.5)^a^	44,840 (4.2)^b^	
20–34	906,164 (79.4)^a^	861,148 (79.8)^b^	
≥ 35	182,934 (16.1)^a^	172,535 (16.0)^b^	
**Gestational Age categories** (weeks+days)			< 0.001
≥ 37 week	1,015,624 (88.98)^a^	960,095 (89.01)^a^	
32 + 0–36 + 6	111,469 (9.77)^a^	105,557 (9.79)^a^	
28 + 0–31 + 6	9929 (0.87)^a^	9066 (0.84)^b^	
22 + 0–27 + 6	4363 (0.38)^a^	3811 (0.35)^b^	
**Mode of delivery**			< 0.001
Spontaneous vaginal delivery	482,703 (42.3)^a^	428,957 (39.8)^b^	
Total CS rate	658,682 (57.7)^a^	649,572 (60.2) ^b^	
**Primary versus Previous CS**			< 0.001
Spontaneous vaginal delivery	477,043 (41.8) ^a^	424,061 (39.3) ^b^	
Spontaneous vaginal delivery (Previous CS history)	5660 (0.5) ^a^	4896 (0.5) ^b^	
Primary CS	329,051 (28.8) ^a^	331,932 (30.8) ^b^	
Repeated CS	329,631 (28.9) ^a^	317,640 (29.4) ^b^	
**Parity**			0.138
Nulliparity	419,758 (36.8)	397,677 (36.9)	
Multiparity	721,627 (63.2)	680,852 (63.1)	
**Pregnancy**			< 0.001
Singleton	1,103,298 (96.7) ^a^	1,044,879 (96.9) ^b^	
Multiple	38,087 (3.3) ^a^	33,650 (3.1) ^b^	
**Presentation**			< 0.001
Cephalic	1,056,275 (92.5) ^a^	996,902 (92.4) ^b^	
Breech	64,227 (5.6) ^a^	61,093 (5.7) ^a^	
Abnormal lie	20,883 (1.8) ^a^	20,534 (1.9) ^b^	
**Onset of labor**			< 0.001
Spontaneous	1,012,234 (88.7) ^a^	965,862 (89.6) ^b^	
Induced	129,151 (11.3) ^a^	112,667 (10.4) ^b^	
**Health service category**			< 0.001
Public hospital	555,120 (48.6) ^a^	490,923 (45.5) ^b^	
Private hospital	507,708 (44.5) ^a^	518,075 (48.0) ^b^	
University hospital	78,557 (6.9) ^a^	69,531 (6.4) ^b^	
**Infant sex**			0.785
Female	555,992 (48.7)	525,176 (48.7)	
Male	585,393 (51.3)	553,353 (51.3)	
**Birth weight**			< 0.001
≥ 2500 g	1,050,314 (92.02) ^a^	995,890 (92.34) ^b^	
1500- < 2500 g	78,946 (6.92) ^a^	71,796 (6.66) ^b^	
1000- < 1500 g	7641 (0.69) ^a^	6887 (0.64) ^b^	
< 1000 g	4484 (0.40) ^a^	3956 (0.37) ^b^	
**Province of residence (NUTS-12)**			< 0.001
Istanbul Region	197,743 (17.3) ^a^	184,540 (17.1) ^b^	
West Marmara Region	37,208 (3.3)	35,638 (3.3)	
Aegean Region	116,090 (10.2)	110,179 (10.2)	
East Marmara Region	102,760 (9.0)	96,762 (9.0)	
West Anatolia Region	99,898 (8.8)	93,843 (8.7)	
Mediterranean Region	143,862 (12.6)	136,206 (12.6)	
Central Anatolia Region	50,404 (4.4) ^a^	47,365 (4.1) ^b^	
West Black Sea Region	47,704 (4.2)	44,175 (4.1)	
East Black Sea	28,563 (2.5)	26,843 (2.5)	
Northeast Anatolia Region	39,017 (3.4)	36,715 (3.4)	
Central East Anatolia Region	72,440 (6.3)	68,005 (6.3)	
Southeast Anatolia Region	205,696 (18.0) ^a^	198,258 (18.4) ^b^	

^ab^Values having different letters in the same row is statistically significant; < 0.05

^*^Discrepancies between the denominator for maternal age categories included in the study are due to missing data (*n* = 12,293, 1.1% in 2019, and *n* = 6, 0% in 2020)

Deliveries reduced in public and university facilities and increased in private facilities in 2020 compared to 2019. There were fewer multiple pregnancies (3.1%) in 2020 than that (3.3%) in 2019. The rate of spontaneous labor was higher in 2020 compared to that in 2019. The rate of CS delivery among hospital deliveries in 2020 was found to be significantly higher than that in 2019. The primary CS rate was 30.8% in 2020, which was significantly higher than 28.8% in 2019. Overall, we identified 11% preterm births, similar in 2020 compared to 2019. However, there was a significant reduction in very and extremely preterm births in 2020. The percentage of LBW in 2020 was lower than in 2019.

Regarding the NUTS-1 regions, total births were significantly higher in Southeast Anatolia, and lower in Istanbul and Central Anatolia regions in 2020 compared to 2019.

The distribution of CS rates according to the TGCS was given in Table 2. CS rate in women with a single, cephalic presentation at term, and a previous CS, the largest individual contributor to the overall CS increased from 98.1 to 98.3%. The next largest contributor was nulliparous women with a term, singleton, cephalic-presenting pregnancy in spontaneous labor, and the CS rate in this group increased from 52.4 to 57.8%. The largest group in the obstetric population, “multiparous women with no previous CS, term, singleton, cephalic presenting pregnancy in spontaneous labor”, had the lowest CS rate (14.4% in 2019, 16.2% in 2020). CS rates in all groups of the Robson TGCS except breech deliveries (Grup 6 and 7) were higher in 2020 than that in 2019.

**Table 2:** Distribution of CS rates according to the 10-group Robson classification system

	Pre-pandemic, 2019 (Total birth = 1,141,385, total CS = 658,682, CS rate = 57.7%)			During-pandemic, 2020 (total birth = 1,078,529, total CS = 649,572, CS rate = 60.2%)			*p*
	Total birth n, %	CS rate n, %	Contribution made by each group to the total CS rate, %	Total birth n, %	CS rate n, %	Contribution of each to total CS rate, %	CS rate; 2019 vs. 2020
1. Nulliparous, single cephalic, ≥37 weeks spontaneous labor	286,498 25.1%	150,105 52.4%	13.2	275,546 25.5%	159,345 57.8%	14.8	< 0.001
2. Nulliparous, single cephalic, ≥37 weeks. Induced or CS before labor	45,696 4.0%	21,762 47.6%	1.9	39,825 3.7%	19,788 49.7%	1.8	< 0.001
3. Multiparous (excluding previous CS), single cephalic, ≥37 weeks. Spontaneous labor	294,844 25.8%	42,575 14.4%	3.7	272,994 25.3%	44,118 16.2%	4.1	< 0.001
4. Multiparous (excluding previous CS), single cephalic, ≥37 weeks. Induced or CS before labor	39,728 3.5%	6854 17.3%	0.6	36,102 3.3%	6494 18.0%	0.6	< 0.001
5. Previous CS, single cephalic, ≥37 weeks	271,131 23.8%	266,110 98.1%	23.3	260,971 24.2%	256,549 98.3%	23.8	< 0.001
6. All nulliparous breeches	28,900 2.5%	28,451 98.4%	2.5	28,477 2.6%	28,058 98.5%	2.6	0.420
7. All multiparous breeches (including previous CS)	28,471 2.5%	27,569 96.8%	2.4	26,463 2.5%	25,574 96.6%	2.4	0.207
8. All multiple pregnancies (including previous CS)	38,087 3.3%	35,438 93.0%	3.1	33,650 3.1%	31,605 93.9%	2.9	< 0.001
9. All abnormal lies (including previous CS)	19,328 1.7%	19,210 99.4%	1.7	19,303 1.8%	18,362 95.1%	1.7	< 0.001
10. All single cephalic, ≤36 weeks (including previous CS)	88,702 7.8%	60,608 68.3%	5.3	85,198 7.9%	59,682 70.1%	5.5	< 0.001

### CS rates for women with singleton, cephalic pregnancies without previous CS history

CS rates for women with singleton, cephalic pregnancies without previous CS history were 34.5% in 2019 and 38.0% in 2020 (Table 3). After controlling for NUTS region, maternal age, parity, gestational week, birth weight, the onset of labor, and infant sex with year and period interaction, CS rates were found to be significantly increased during the 3rd and 4th periods with respect to the pre-pandemic first period. CS rates for women with singleton, cephalic pregnancies without previous CS history in 2019 and 2020 were 52.7 and 57.5% for nulliparous pregnancies and 16.1 and 17.7% for multiparous pregnancies, respectively (Table 3). When nulliparous women with singleton and cephalic pregnancies were enrolled, the 3rd period had a considerably higher odds ratio for CS and the 5th period had lower odds ratio compared to the pre-pandemic period with year and period interaction after adjusting confounding factors. However, multiparous women with singleton and cephalic pregnancies without previous CS history were showed a significantly higher odds ratio for CS in the 3rd, 4th, and 5th period compared to the pre-pandemic period after adjusting confounding factors with year and period interaction.

**Table 3:** Data on singleton, cephalic pregnancies without previous CS history

	CS births, 2019 n (%)	CS births, 2020 n(%)	Model 1 Adjusted OR (95% CI)	*p*	Model 2 Adjusted OR (95% CI)	*P*
**Singleton, cephalic pregnancies without previous CS history**						
Period 1 Jan, Feb	37,741 (33.8)	39,046 (36.6)	1.00		1.00	
Period 2 March, May	60,640 (34.2)	61,295 (37.4)	1.02 (1.00, 1.04)	0.120	1.02 (1.00, 1.05)	0.118
Period 3 June, August	68,129 (34.3)	72,138 (38.5)	1.06 (1.04, 1.09)	0.000	1.07 (1.05, 1.10)	0.000
Period 4 September, October	42,647 (35.1)	44,821 (39.1)	1.05 (1.03, 1.08)	0.000	1.05 (1.02, 1.08)	0.001
Period 5 November, December	40,173 (35.5)	39,794 (38.3)	1.0 (0.97, 1.02)	0.915	1.01 (0.98, 1.04)	0.655
Total	249,330 (34.5)	257,094 (38.0)				
**Nulliparous, singleton, cephalic pregnancies without previous CS history**						
Period 1 Jan, Feb	28,152 (52.2)	29,398 (57.0)	1.00		1.00	
Period 2 March, May	46,058 (52.9)	47,190 (57.9)	1.01 (0.98, 1.04)	0.643	1.02 (0.99, 1.05)	0.210
Period 3 June, August	53,561 (50.9)	56,664 (56.6)	1.03 (1.00, 1.06)	0.038	1.05 (1.02, 1.09)	0.001
Period 4 September, October	33,277 (53.2)	35,160 (58.3)	1.01 (0.98, 1.05)	0.407	1.03 (0.99, 1.06)	0.165
Period 5 November, December	30,527 (55.6)	30,077 (58.5)	0.93 (0.90, 0.96)	0.000	0.96 (0.93, 1.00)	0.026
Total	191,575 (52.7)	198,489 (57.5)				
**Multiparous, singleton, cephalic pregnancies without previous CS history**						
Period 1 Jan, Feb	9589 (16.6)	9648 (17.5)	1.00		1.00	
Period 2 March, May	14,582 (16.2)	14,105 (17.1)	1.01 (0.97, 1.05)	0.674	1.02 (0.98, 1.06)	0.465
Period 3 June, August	14,568 (15.6)	15,474 (17.7)	1.10 (1.06, 1.15)	0.000	1.10 (1.05, 1.14)	0.000
Period 4 September, October	9370 (15.9)	9661 (17.7)	1.07 (1.03, 1.12)	0.001	1.07 (1.02, 1.12)	0.003
Period 5 November, December	9646 (16.6)	9717 (18.5)	1.08 (1.03, 1.12)	0.001	1.08 (1.03, 1.13)	0.001
Total	57,755 (16.1)	58,605 (17.7)				

Model 1: Year and period interaction were evaluated

Model 2: in addition to model 1, controlled for NUTS region, maternal age, parity, gestational week, birth weight, onset of labor, and infant sex

When NUTS regions of Turkey were analyzed separately with Model 2, CS rates showed different features in regions (Supp. Table 1). Compared to the 1st period (pre-pandemic) İstanbul and Central Anatolia regions had lower odds for CS rates in the 5th period (the second wave), Southeast Anatolia Region had lower odds for CS rates in all pandemic periods. However, higher odds for CS rates were observed in all pandemic periods in Mediterranean and West Black Sea Regions, in the 2nd, 3rd and 4th period in Aegean Region, in the 3rd, 4th, and 5th period in West Anatolia Region, and in the 3rd and 4th period in East Marmara Region, in Period 3 in Northeast Anatolia Region. CS rates in the other three regions (West Marmara Region, East Black Sea, Central East Anatolia) did not differ according to pandemics.

### The preterm rate in women with singleton births

**Fig. 1:**
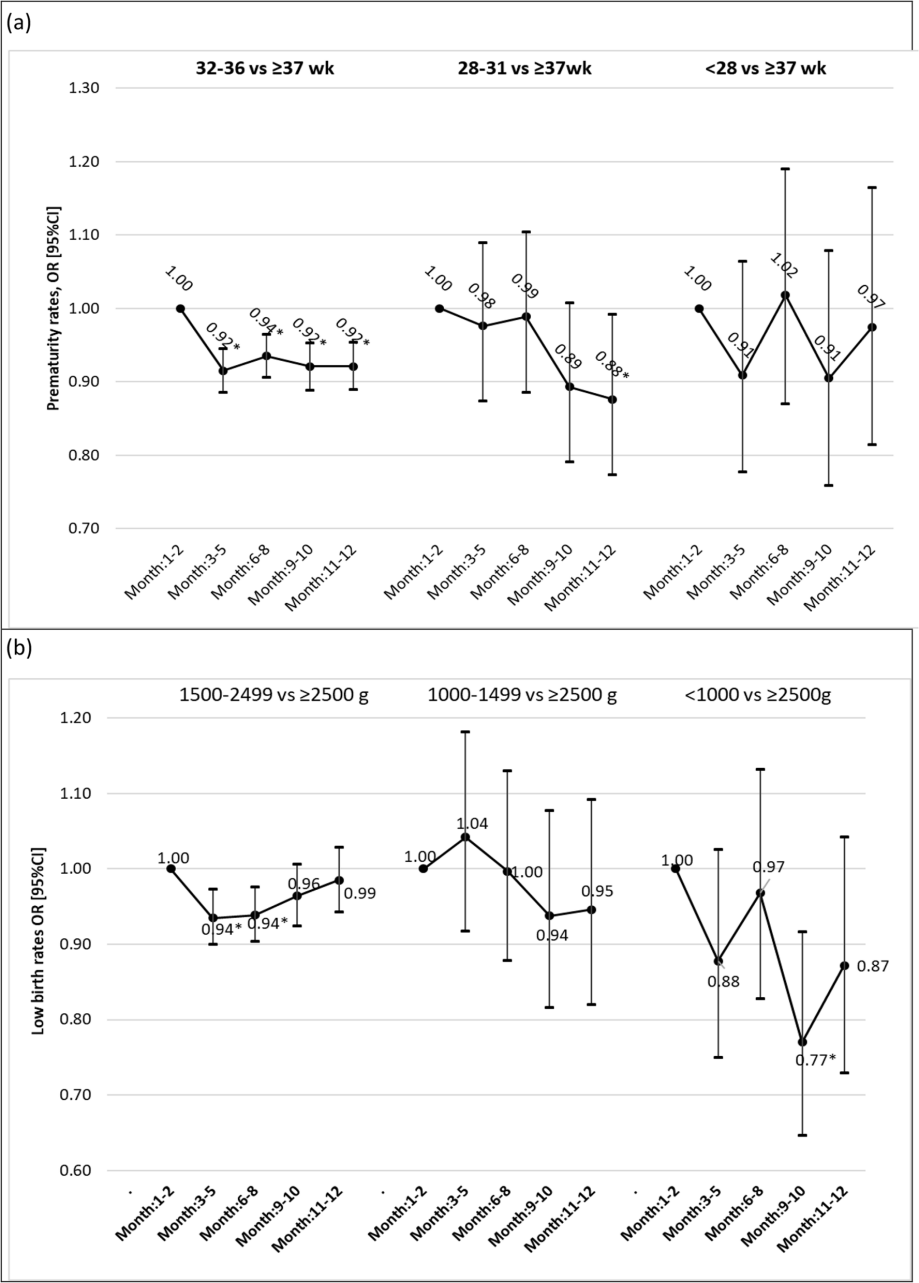
**a**. Differences in degrees of preterm rates compared to term births during pandemic periods from pre-pandemic periods [Estimated for year interaction (2020 vs 2019), and controlled for NUTS region, maternal age, mode of delivery, parity, onset of labor, presentation birth weight and infant sex in singleton pregnancies (Model 2); *p < 0.05]. **b** Differences in degrees of LBW rates compared to normal weight births during pandemic periods from pre-pandemic periods [Estimated for year interaction (2020 vs 2019), and controlled for NUTS region, maternal age, birth type, parity, onset of labor, presentation, and infant sex in singleton pregnancies; *p < 0.05]

The preterm rate in women with singleton births was 9.0% in 2019, and 9.1% in 2020. Multivariable analysis showed a significant reduction in odds ratio between 6 to 8% at all time periods with respect to the pre-lockdown period and the same period in the previous year after adjusting for NUTS region, maternal age, parity, mode of delivery, the onset of labor, presentation, birth weight, and infant sex with year and period interaction (Table 4). Very preterm births were reduced in the 5th period, and unchanged at other periods. No change was observed in extremely preterm births at any time period (Fig. 1a). The preterm rate in singleton spontaneous births was 6.0% in 2019, and 6.1% in 2020. The preterm rate in singleton induced or CS births was 10.8% in 2019, and 10.7% in 2020. Preterm rates showed similar changes in both spontaneous singleton preterm births and medical (induced or CS) singleton preterm births during the pandemic period when confounding factors adjusted (Table 4). Moderate to late singleton preterm births were reduced at all periods. Very preterm births were reduced in the second lockdown period, and unchanged at other periods. No change was observed in extremely preterm births at any time period.

**Table 4:** Changes in singleton preterm births according to periods and years

	Preterm births, 2019 n(%)	Preterm births, 2020 n(%)	Model 1 Adjusted OR (95% CI)	*p*	Model 2 Adjusted OR (95% CI)	*P*
**Singleton preterm births**						
Period 1 Jan, Feb	14,673 (8.4)	15,059 (9.0)	1.00		1.00	
Period 2 March, May	24,301 (8.9)	22,336 (8.8)	0.92 (0.89, 0.95)	< 0.001	0.92 (0.89, 0.95)	< 0.001
Period 3 June, August	26,888 (9.0)	26,331 (9.2)	0.95 (0.92, 0.98)	0.001	0.94 (0.91, 0.97)	< 0.001
Period 4 September, October	16,997 (9.2)	16,285 (9.2)	0.93 (0.90, 0.96)	< 0.001	0.92 (0.89, 0.95)	< 0.001
Period 5 November, December	16,085 (9.2)	14,791 (9.2)	0.92 (0.89, 0.95)	< 0.001	0.92 (0.89, 0.95)	< 0.001
**Total**	98,944. (9.0)	94,802 (9.1)				
**Spontan singleton preterm births**						
Period 1 Jan, Feb	3515 (5.4)	3684 (6.1)	1.00		1.00	
Period 2 March, May	5863 (5.7)	5338 (5.8)	0.90 (0.85, 0.96)	0.001	0.90 (0.85, 0.96)	0.001
Period 3 June, August	6998 (6.0)	6391 (6.2)	0.92 (0.86, 0.97)	0.004	0.91 (0.86, 0.97)	0.002
Period 4 September, October	4498 (6.4)	3923 (6.3)	0.87 (0.82, 0.93)	< 0.001	0.86 (0.81, 0.92)	< 0.001
Period 5 November, December	4167 (6.4)	3641 (6.4)	0.88 (0.83, 0.94)	< 0.001	0.87 (0.82, 0.93)	< 0.001
Total	25,041 (6.0)	22,977 (6.1)				
**Induced or ceserean singleton preterm births**						
Period 1 Jan, Feb	11,158 (10.2)	11,375 (10.7)	1.00		1.00	
Period 2 March, May	18,438 (10.9)	16,998 (10.5)	0.92 (0.89, 0.96)	< 0.001	0.93 (0.89, 0.96)	< 0.001
Period 3 June, August	19,890 (10.9)	19,940 (10.8)	0.95 (0.91, 0.98)	0.002	0.95 (0.92, 0.98)	0.004
Period 4 September, October	12,499 (10.9)	12,362 (10.7)	0.93 (0.90, 0.97)	< 0.001	0.94 (0.90, 0.97)	0.001
Period 5 November, December	11,918 (11.0)	11,150 (10.7)	0.93 (0.89, 0.97)	< 0.001	0.94 (0.90, 0.97)	0.001
Total	73,903 (10.8)	71,825 (10.7)				

Model 1: Year and time period interaction were evaluated

Model 2: in addition to model 1 controlled for NUTS region, maternal age, parity, mode of delivery, onset of labor, presentation, birth weight, and infant sex

### The LBWs rate in women with singleton births

When only singleton births were considered, LBW rates were 6.1 and 5.9% in 2019, and 2020 respectively. There was a significant reduction in LBW rates in all periods except the 5th period during the pandemic after controlling for NUTS region, maternal age, parity, mode of delivery, the onset of labor, presentation, gestational age, and infant sex with year and period interaction (Table 5). VLBW births were unchanged, and ELBW births were lower in the 4th period compared to the 1st period (Fig. 1b). LBW rates among preterm births were 41.7% in 2019, and 41.2% in 2020. LBW rates among term births were 2.6% in 2019, and 2.4% in 2020.

**Table 5:** Changes in LBW rates among singleton births according to periods and years

	LBW births, 2019 n(%)	LBW births, 2020 n(%)	Model 1 Adjusted OR (95% CI)	*p*	Model 2 Adjusted OR (95% CI)	*P*
**LBWs**						
Period 1 Jan, Feb	10,266 (5.9)	9958 (6.0)	1.00		1.00	
Period 2 March, May	16,892 (6.2)	14,993 (5.9)	0.94 (0.91, 0.98)	0.002	0.94 (0.91, 0.98)	0.001
Period 3 June, August	18,327 (6.2)	17,023 (6.0)	0.96 (0.92, 0.99)	0.012	0.95 (0.91, 0.98)	0.002
Period 4 September, October	11,216 (6.1)	10,509 (5.9)	0.96 (0.93, 1.00)	0.063	0.95 (0.92, 0.99)	0.015
Period 5 November, December	10,252 (5.9)	9441 (5.8)	0.98 (0.94, 1.02)	0.385	0.98 (0.94, 1.02)	0.253
Total	66,953 (6.1)	61,924 (5.9)				

Model 1: Year and time period interaction were evaluated

Model 2: in addition to model 1 controlled for NUTS region, maternal age, parity, mode of delivery, onset of labor, presentation, gestational age, and infant sex

## Discussion

Our study based on nationwide data from Turkey indicated differences in perinatal outcomes due to pandemic; higher rates for CS, lower rates for preterm births, and LBW. Deliveries reduced in public and university facilities and increased in private facilities during the pandemic period compared to the pre-pandemic period.

Total deliveries were lower during the first and second waves but were higher during the other periods. A reduction of births during the second wave; approximately 9 months after the time of the strongest effects of the pandemic could be interpreted as a failure to conceive due to fear of infection, economic uncertainty, increased child-rearing demands in a time of the pandemic, and reduced social support [4, 5].

Furthermore, our findings showed fewer multiple pregnancies in the pandemic period than in the pre-pandemic period. One possible explanation might be reduced assisted reproductive technology demands in a time of the pandemic.

Another important finding of our study was a change in maternity care service provision. Turkey implemented Transformation in Health program in 2003, and social insurance institution, state pension fund, and self-employed pension fund were united under Social Security Institution. Patients pay a fee difference directly to the private health institutions, where a certain amount of the treatment is paid by the Social Security Institution directly to the private institution. All insured and uninsured individuals in the country including those who were qualified as underprivileged were provided with a universal health insurance [28]. Overall, users of private facilities increased during the pandemic period compared to the pre-pandemic period. Furthermore, similar to previous studies CS rates were high among women cared for in the private sector [29, 30]. This increased utilization of private care facilities during the pandemic may be due to the fact that the maternity care services in these facilities became more available and accessible since the public hospitals were more crowded. To ensure physical distancing in crowded settings, maternity care service utilization might have shifted to private facilities.

In Turkey, CS rates increased from 57.7% in 2019, pre-pandemic period to 60.2% in 2020. The optimal CS rates have been recommended as 10–15% by World Health Organisation [31]. Despite this recommendation, the proportion of CS rates in Turkey rose from 21% in 2003 to 52% in 2018 [23]. Turkey passed a regulation entitled “Physician Unit Performance Coefficient” in Jan 2013 to reduce primary CS rates, restricting CS births to medical necessity [32]. We used Robson 10 group classification which has been endorsed by WHO as the global standard for monitoring, assessing and comparing CS rates [33]. Approximately 29.6% of the population had a history of previous CS in Turkey, which was higher than the reported average globally [33]. No published study was found that analyzed changes in CS rates during the pandemic with the Robson 10 group classification. There was a significant increase in the frequencies of CS for all groups from 2019 to 2020 except breech presentations (group 6–7) and abnormal lies (group 9) in Turkey. The frequencies of CS for abnormal lies (group 9) were lower in 2020 compared to 2019. However, CS rate in breech deliveries were unchanged during the pandemic. On the other hand, the fact that the pandemic period is going with changes in the way of lockdown and opening requires making separate analyzes according to the periods. Considering the increase in trend of CS rates we performed the multivariate analysis with year and period interaction. Indeed, the analysis showed a higher rate for CS during the 3rd and 4th periods of the pandemic period with respect to the pre-pandemic 1st period after adjusting confounding factors and year-period interaction. As an indirect impacts of the pandemic on the healthcare system, the preference of private hospitals might have contributed to the increased prevalence of CS deliveries. In addition, the change in CS rates differed according to the NUTS regions. By regions, the percentage of women who had CS was highest in Mediterranean region, followed by Aegean, West Marmara, and Black Sea regions. Northeast Anatolia region, followed by Central East Anatolia and Southeast Anatolia had the lowest CS rates. Western part of Turkey can be considered as the most urbanized, industrialized and the wealthiest regions. Mediterranean region can also be considered as the urban regions where growing industrial centers reside. On the other hand, eastern regions are the least urbanized and socioeconomically disadvantaged. Inappropriate use of CS might explain the observed variations across regions, but that was beyond the scope of our study. These regional variations should be explored further in future studies.

Despite similar preterm birth rates (11%) in 2019 and 2020, when singleton pregnancies were considered, multivariable analyses showed lower preterm proportions during all time periods with respect to the pre-pandemic period. Studies evaluating preterm births yielded mixed results [13, 24, 34]. In Italy, a uniform reduction in preterm births was confined to moderate to late preterm births [35]. As suggested by the previous studies, lower preterm rates during pandemic might be due to lifestyle changes including cessation of work, increased hygiene measures, social distancing resulting in fewer infections by common pathogens, less air pollution [4, 14, 35]. Yet, a recent analysis concluded that due to methodologic issues, and the discrepancies in results there is insufficient evidence to conclude that there was a consistent reduction in preterm birth associated with the lockdowns [15]. Further studies should be performed to clarify the underlying mechanisms of this reduction.

The percentage of LBW was lower in 2020 compared to 2019 (7.7% vs. 8.0%). LBW rates and ELBW births changed according to lockdown periods. VLBW births were similar in periods. Reduction in VLBW and ELBW infants was observed in Ireland from January to April 2020 compared to historical data coinciding with the first lockdown in the Ireland study [36]. Similarly, mean birth weight was reported to be significantly higher and the rates of LBW, VLBW, and ELBW were significantly lower during the first COVID 19 lockdown phase in Austria [34]. Since we did not have stillbirth or miscarriage data, we could not comment on early pregnancy or late fetal loss. Socio-environmental alterations such as reduced exposure to infectious agents, reduced work-related stress, resting at home, reduced air pollution due to lockdown measures have been suggested as the potential underlying mechanism [4, 14, 35].

### Strengths and limitations

This study is the first one in the world to examine the impact of different periods (five periods) of the pandemic on perinatal outcomes. Most studies only reported the changes in the first lockdown period and followed 3–6 months [24, 34–36]. This study fully shows the situation in the society in Turkey since all the national data for hospital deliveries were examined without taking any samples. Also, there is no change in the data collection system of the Ministry of Health in 2019 and 2020. The strengths of the study included nationally representative data, a long time frame, and a comparable period in 2019. The sample design allowed estimations for the regional levels in Turkey as well.

Nonetheless, some limitations need to be considered. We had no information on CS indications, pregnancy complications, full demographics of the women such as education, socio-economic status, stillbirth rates, and prevalence of COVID-19 among the study population. Since our main objective was not to explore the mortality rates but rather birth-related outcomes, we did not have the mortality data. In addition, since data were retrieved from the registry, records that were not registered might be missing. We only analyzed hospital deliveries. However, 3% of deliveries occurred at home in 2019 [30]. As a strength, we analysed all national data including 2 years; 2019–2020.

## Conclusions

Similar to previous studies [34, 37], our overall findings showed no negative effect of the pandemic on neonatal outcomes such as preterm and newborn weight. Neonatal outcomes such as preterm birth and newborn weight improved possibly due to the indirect effects of the pandemic. On the other hand, CS rates were increased and maternity care service utilization shifted to private facilities. It is necessary to evaluate changes in perinatal outcomes with further studies after the pandemic.

## Supplementary Information

**Additional file 1: Supp. Table 1.** Cesarean births on singleton, cephalic pregnancies without previous CS history in province of residence (NUTS region) of Turkey.

## Data Availability

The data sets are available from General Directorate of Public Health, Ministry of Health on request. The datasets analyzed are not publicly available since they belonged to the Ministry of Health.

## Abbreviations

CS: Cesarean delivery
LBW: Low birth weight
NUTS: Nomenclature of Territorial Units for Statistics
VLBW: Very low birth weight
ELBW: Extremely low birth weight
TGCS: 10-group classification system
OR: Odds ratio
CI: Confidence interval
