# Supplementary material for: Effects of the COVID-19 pandemic on perinatal outcomes: a retrospective cohort study from Turkey
Source: BMC Pregnancy Childbirth. 2022 Jan 20;22:51. doi: 10.1186/s12884-021-04349-5 (PMC8772535; doi:10.1186/s12884-021-04349-5)
Supplement: Supplementary file 1 — Additional file 1: Supp. Table 1. Cesarean births on singleton, cephalic pregnancies without previous CS history in province of residence (NUTS region) of Turkey. [file 12884_2021_4349_MOESM1_ESM.docx]

Supplementary Table

**Table. Cesarean births on singleton, cephalic pregnancies without previous CS history in province of residence (NUTS region) of Turkey**

|  | CS births. 2019 | | CS births. 2020 | |  | 95% CI | |  |  | 95% CI | |  |
| --- | --- | --- | --- | --- | --- | --- | --- | --- | --- | --- | --- | --- |
|  | N | % | n | % | AOR* | Lower | Upper | p | AOR** | Lower | Upper | p |
| Istanbul Region |  |  |  |  |  |  |  |  |  |  |  |  |
| Period 1 | 8010 | 40.7 | 8504 | 44.4 | 1.00 |  |  |  | 1.00 |  |  |  |
| Period 2 | 12512 | 42.0 | 12839 | 45.6 | 1.00 | 0.95 | 1.05 | 0.927 | 0.99 | 0.93 | 1.05 | 0.694 |
| Period 3 | 13862 | 40.6 | 14497 | 44.6 | 1.01 | 0.96 | 1.06 | 0.657 | 1.04 | 0.98 | 1.10 | 0.218 |
| Period 4 | 8879 | 41.9 | 9241 | 45.0 | 0.97 | 0.92 | 1.03 | 0.355 | 0.98 | 0.92 | 1.04 | 0.500 |
| Period 5 | 8811 | 43.4 | 8013 | 44.0 | 0.88 | 0.83 | 0.93 | <0.001 | 0.90 | 0.85 | 0.96 | 0.001 |
| Total | 52074 | 41.6 | 53094 | 44.8 |  |  |  |  |  |  |  |  |
| West Marmara Region |  |  |  |  |  |  |  |  |  |  |  |  |
| Period 1 | 1571 | 44.0 | 1605 | 46.3 | 1.00 |  |  |  | 1.00 |  |  |  |
| Period 2 | 2580 | 44.7 | 2612 | 48.1 | 1.05 | 0.93 | 1.18 | 0.468 | 0.99 | 0.87 | 1.14 | 0.905 |
| Period 3 | 2842 | 43.9 | 3078 | 48.8 | 1.11 | 0.99 | 1.24 | 0.088 | 1.09 | 0.95 | 1.24 | 0.219 |
| Period 4 | 1783 | 45.4 | 1861 | 49.7 | 1.08 | 0.95 | 1.23 | 0.238 | 1.11 | 0.96 | 1.29 | 0.158 |
| Period 5 | 1615 | 45.5 | 1685 | 51.0 | 1.13 | 0.99 | 1.30 | 0.067 | 1.13 | 0.97 | 1.31 | 0.123 |
| Total | 10391 | 44.6 | 10841 | 48.7 |  |  |  |  |  |  |  |  |
| Aegean Region |  |  |  |  |  |  |  |  |  |  |  |  |
| Period 1 | 4543 | 41.7 | 4785 | 45.2 | 1.00 |  |  |  | 1.00 |  |  |  |
| Period 2 | 7326 | 42.6 | 7403 | 46.8 | 1.03 | 0.96 | 1.10 | 0.463 | 1.09 | 1.01 | 1.17 | 0.034 |
| Period 3 | 8321 | 42.8 | 8836 | 48.0 | 1.07 | 1.00 | 1.14 | 0.062 | 1.10 | 1.02 | 1.19 | 0.012 |
| Period 4 | 5158 | 44.8 | 5527 | 49.9 | 1.06 | 0.99 | 1.15 | 0.110 | 1.09 | 1.00 | 1.19 | 0.043 |
| Period 5 | 4844 | 44.5 | 4895 | 48.6 | 1.02 | 0.94 | 1.10 | 0.658 | 1.05 | 0.97 | 1.15 | 0.243 |
| Total | 30192 | 43.2 | 31446 | 47.7 |  |  |  |  |  |  |  |  |
| Mediterranean Region |  |  |  |  |  |  |  |  |  |  |  |  |
| Period 1 | 5149 | 40.7 | 5059 | 42.3 | 1.00 |  |  |  | 1.00 |  |  |  |
| Period 2 | 7643 | 40.7 | 7660 | 45.3 | 1.13 | 1.06 | 1.21 | <0.001 | 1.14 | 1.05 | 1.22 | 0.001 |
| Period 3 | 8911 | 41.4 | 9214 | 45.7 | 1.12 | 1.05 | 1.19 | 0.001 | 1.12 | 1.04 | 1.20 | 0.003 |
| Period 4 | 5652 | 41.5 | 6035 | 46.4 | 1.14 | 1.07 | 1.23 | <0.001 | 1.11 | 1.02 | 1.20 | 0.011 |
| Period 5 | 5443 | 41.9 | 5503 | 45.7 | 1.10 | 1.02 | 1.18 | 0.013 | 1.10 | 1.01 | 1.19 | 0.029 |
| Total | 32798 | 41.2 | 33471 | 45.2 |  |  |  |  |  |  |  |  |
| West Anatolia Region |  |  |  |  |  |  |  |  |  |  |  |  |
| Period 1 | 3674 | 35.1 | 3486 | 36.4 | 1.00 |  |  |  | 1.00 |  |  |  |
| Period 2 | 5952 | 35.3 | 5804 | 37.7 | 1.05 | 0.98 | 1.13 | 0.201 | 1.04 | 0.96 | 1.12 | 0.402 |
| Period 3 | 6290 | 33.8 | 6793 | 38.4 | 1.16 | 1.08 | 1.25 | <0.001 | 1.20 | 1.11 | 1.30 | <0.001 |
| Period 4 | 3950 | 35.8 | 4227 | 40.1 | 1.14 | 1.05 | 1.24 | 0.001 | 1.17 | 1.08 | 1.28 | <0.001 |
| Period 5 | 3608 | 35.2 | 3706 | 40.4 | 1.19 | 1.09 | 1.29 | <0.001 | 1.26 | 1.15 | 1.38 | <0.001 |
| Total | 23474 | 34.9 | 24016 | 38.5 |  |  |  |  |  |  |  |  |
| East Marmara Region |  |  |  |  |  |  |  |  |  |  |  |  |
| Period 1 | 3975 | 39.3 | 4135 | 42.0 | 1.00 |  |  |  | 1.00 |  |  |  |
| Period 2 | 6375 | 40.2 | 6447 | 43.8 | 1.04 | 0.97 | 1.12 | 0.312 | 1.08 | 1.00 | 1.17 | 0.063 |
| Period 3 | 7207 | 39.8 | 7719 | 44.7 | 1.09 | 1.02 | 1.17 | 0.014 | 1.14 | 1.05 | 1.23 | 0.001 |
| Period 4 | 4482 | 40.8 | 4643 | 45.5 | 1.09 | 1.00 | 1.17 | 0.042 | 1.10 | 1.01 | 1.20 | 0.032 |
| Period 5 | 4169 | 41.3 | 4076 | 44.3 | 1.01 | 0.93 | 1.09 | 0.809 | 1.04 | 0.95 | 1.14 | 0.430 |
| Total | 26208 | 40.2 | 27020 | 44.1 |  |  |  |  |  |  |  |  |
| West Black Sea Region |  |  |  |  |  |  |  |  |  |  |  |  |
| Period 1 | 1845 | 42.3 | 1780 | 43.7 | 1.00 |  |  |  | 1.00 |  |  |  |
| Period 2 | 3094 | 42.3 | 3089 | 46.6 | 1.12 | 1.01 | 1.25 | 0.035 | 1.13 | 1.00 | 1.28 | 0.045 |
| Period 3 | 3473 | 42.6 | 3796 | 49.1 | 1.23 | 1.11 | 1.37 | <0.001 | 1.35 | 1.20 | 1.52 | <0.001 |
| Period 4 | 2077 | 42.3 | 2282 | 50.5 | 1.32 | 1.17 | 1.48 | <0.001 | 1.37 | 1.20 | 1.57 | <0.001 |
| Period 5 | 1809 | 43.0 | 1908 | 48.8 | 1.19 | 1.06 | 1.35 | 0.005 | 1.27 | 1.11 | 1.46 | 0.001 |
| Total | 12298 | 42.5 | 12855 | 47.8 |  |  |  |  |  |  |  |  |
| East Black Sea |  |  |  |  |  |  |  |  |  |  |  |  |
| Period 1 | 1150 | 42.7 | 1148 | 45.1 | 1.00 |  |  |  | 1.00 |  |  |  |
| Period 2 | 2030 | 45.7 | 1946 | 48.8 | 1.03 | 0.90 | 1.18 | 0.676 | 1.05 | 0.90 | 1.23 | 0.551 |
| Period 3 | 2341 | 45.5 | 2181 | 47.2 | 0.97 | 0.85 | 1.11 | 0.675 | 1.04 | 0.89 | 1.21 | 0.613 |
| Period 4 | 1368 | 44.7 | 1385 | 47.7 | 1.03 | 0.88 | 1.19 | 0.745 | 1.09 | 0.92 | 1.29 | 0.312 |
| Period 5 | 1207 | 44.1 | 1150 | 48.4 | 1.08 | 0.93 | 1.27 | 0.313 | 1.10 | 0.93 | 1.31 | 0.274 |
| Total | 8096 | 44.8 | 7810 | 47.5 |  |  |  |  |  |  |  |  |
| Central Anatolia Region |  |  |  |  |  |  |  |  |  |  |  |  |
| Period 1 | 1476 | 29.4 | 1578 | 35.1 | 1.00 |  |  |  | 1.00 |  |  |  |
| Period 2 | 2598 | 31.0 | 2535 | 33.0 | 0.85 | 0.76 | 0.94 | 0.003 | 0.90 | 0.80 | 1.02 | 0.086 |
| Period 3 | 2909 | 32.4 | 2989 | 34.9 | 0.86 | 0.78 | 0.96 | 0.006 | 0.94 | 0.84 | 1.05 | 0.269 |
| Period 4 | 1701 | 32.3 | 1777 | 36.5 | 0.93 | 0.83 | 1.05 | 0.243 | 0.96 | 0.84 | 1.09 | 0.504 |
| Period 5 | 1629 | 33.9 | 1531 | 34.7 | 0.80 | 0.71 | 0.90 | <0.001 | 0.84 | 0.74 | 0.96 | 0.011 |
| Total | 10313 | 31.8 | 10410 | 34.7 |  |  |  |  |  |  |  |  |
| Northeast Anatolia Region |  |  |  |  |  |  |  |  |  |  |  |  |
| Period 1 | 826 | 21.3 | 939 | 23.8 | 1.00 |  |  |  | 1.00 |  |  |  |
| Period 2 | 1517 | 21.0 | 1532 | 22.6 | 0.95 | 0.83 | 1.08 | 0.443 | 1.01 | 0.87 | 1.17 | 0.928 |
| Period 3 | 1645 | 20.7 | 1917 | 25.5 | 1.13 | 0.99 | 1.29 | 0.061 | 1.25 | 1.08 | 1.44 | 0.002 |
| Period 4 | 1056 | 22.5 | 1146 | 26.2 | 1.05 | 0.91 | 1.21 | 0.491 | 1.12 | 0.96 | 1.31 | 0.165 |
| Period 5 | 912 | 22.1 | 1033 | 25.8 | 1.06 | 0.92 | 1.23 | 0.422 | 1.15 | 0.98 | 1.36 | 0.086 |
| Total | 5956 | 21.4 | 6567 | 24.7 |  |  |  |  |  |  |  |  |
| Central East Anatolia Region |  |  |  |  |  |  |  |  |  |  |  |  |
| Period 1 | 1493 | 21.3 | 1669 | 23.6 | 1.00 |  |  |  | 1.00 |  |  |  |
| Period 2 | 2700 | 20.3 | 2793 | 23.0 | 1.02 | 0.93 | 1.13 | 0.640 | 0.99 | 0.89 | 1.11 | 0.911 |
| Period 3 | 3139 | 21.4 | 3371 | 25.0 | 1.08 | 0.98 | 1.18 | 0.144 | 0.99 | 0.90 | 1.11 | 0.913 |
| Period 4 | 1809 | 21.7 | 1928 | 25.2 | 1.07 | 0.96 | 1.19 | 0.227 | 0.95 | 0.84 | 1.07 | 0.360 |
| Period 5 | 1639 | 22.1 | 1772 | 26.0 | 1.09 | 0.97 | 1.21 | 0.150 | 0.96 | 0.85 | 1.08 | 0.501 |
| Total | 10780 | 21.2 | 11533 | 24.5 |  |  |  |  |  |  |  |  |
| Southeast Anatolia Region |  |  |  |  |  |  |  |  |  |  |  |  |
| Period 1 | 4029 | 18.9 | 4358 | 21.7 | 1.00 |  |  |  | 1.00 |  |  |  |
| Period 2 | 6313 | 19.5 | 6635 | 21.9 | 0.97 | 0.92 | 1.04 | 0.405 | 0.93 | 0.87 | 0.99 | 0.022 |
| Period 3 | 7189 | 20.2 | 7747 | 23.3 | 1.01 | 0.95 | 1.07 | 0.817 | 0.94 | 0.88 | 1.00 | 0.051 |
| Period 4 | 4732 | 20.6 | 4769 | 22.3 | 0.93 | 0.87 | 1.00 | 0.039 | 0.91 | 0.84 | 0.97 | 0.006 |
| Period 5 | 4487 | 20.6 | 4522 | 22.1 | 0.92 | 0.86 | 0.99 | 0.017 | 0.84 | 0.78 | 0.90 | <0.001 |
| Total | 26750 | 20.0 | 28031 | 22.3 |  |  |  |  |  |  |  |  |

*Model 1: Year and time period interaction were evaluated; **Model 2: Controlled for NUTS region. maternal age. parity. gestational week. birthweight. onset of labor. and infant sex

Period 1 Jan,Feb; Period 2 March-May; Period 3 June-August; Period 4 September-October; Period 5 November-December
